# Classification of Emotions Based on Electrodermal Activity and Transfer Learning - a Pilot Study

**DOI:** 10.2478/joeb-2021-0021

**Published:** 2021-12-30

**Authors:** Fredrik A. Jacobsen, Ellen W. Hafli, Christian Tronstad, Ørjan G. Martinsen

**Affiliations:** 1 Department of Physics, University of Oslo, Oslo Norway; 2 Department of Clinical and Biomedical Engineering, Oslo University Hospital, Oslo Norway

**Keywords:** Machine learning, transfer learning, assessing emotions, skin conductance

## Abstract

This paper describes the development, execution and results of an experiment assessing emotions with electrodermal response measurements and machine learning. With ten participants, the study was carried out by eliciting emotions through film clips. The data was gathered with the Sudologger 3 and processed with continuous wavelet transformation. A machine learning algorithm was used to classify the data with the use of transfer learning and random forest classification. The results showed that the experiment lays a foundation for further exploration in the field. The addition of augmented data strengthened the classification and proved that more data would benefit the machine learning algorithm. The pilot study brought to light several areas to help with the expansion of the study for larger scale assessment of emotions with electrodermal response measurements and machine learning for the benefit of fields like psychology.

## Introduction

The last decades have seen a tremendous progress in medical technology for both diagnostics and treatment. However, this has mainly been limited to somatic medicine and there has not been a similar development within the mental health area. Emotional elicitation, contrary to general arousal research, requires not only physiological measures but also subjective reports. Different emotions may elicit the same amount of general arousal, thus becoming possibly indistinguishable with respect to their autonomous nervous system (ANS) response [1].

A common point of view is that emotions are natural responses that has a biological foundation [2]. Neuroscientific studies suggest that the development of emotion is similar to that of cognition, and is integrated in the neural network together with both cognition and attention [3]. Lindquist et al. note that these cortical networks branch out to the limbic system when emotion is involved. Although the limbic system is involved in the processes of the ANS, researchers are still unsuccessful in recognising a pattern in autonomic activation for each emotion. As a result, the general consensus to date is that all emotions increase autonomic activation, and can be measured as such.

Electrodermal activity (EDA), also called measurements of galvanic skin response, has been utilised for over a century [4]. The changing skin conductance is a direct measurement of sweat gland activity, which is linked to the sympathetic part of the nervous system [5].

The aim of this pilot study was to explore the possibilities in emotion classification from EDA recordings using machine learning and transfer learning as an approach for small data sets.

## Materials and methods

The test subjects consisted of ten 20-30 years old volunteers (five female and five male).

A large database of emotion-eliciting films [6] was used in this project. Having this database allowed us to pick and choose film clips that would have an already well-proven emotion-eliciting effect.

From the database, ten film clips were chosen. The film-clip-selection was limited to six categories of emotions, and neutral, to limit the complexity for the machine learning program and taking into consideration the small number of test-subjects. The seven categories were anger, sadness, fear, disgust, amusement, tenderness and neutral. These clips were edited together to one long sequence with ten seconds of pause at the start, between each clip, and at the end.

Trying to have the least amount of distraction for the test subjects, a room was set up on campus for the trials. The set-up consisted of a large television, a chair for the subject to sit comfortably in, a table to write on, a desk lamp, and a table to relax the non-writing arm, fitted with the skin electrodes. To label the data, each test subject was asked to self-report on what emotion, out of the seven given, was felt strongest for each film clip.

The subject was taken into the room where he/she sat in front of the television. They were then presented with the consent form, which explains the procedures and how the data will be treated afterwards. After the consent form was read and signed, the electrodes were attached for conductance measurements. While the electrodes got some time to stabilize, the subject was presented with the self-report form and we explained how the film was set up and how they should write down their answer during the ten-second pauses in between the clips. Test subjects were told to sit as still as possible during the measurements.

A person from the project was always present in the room during the measurements and took notes if the subject moved a lot, coughed, etc. After the film ended the measurements were immediately stopped to help synchronize the data with the timing of the clips. For the ten-clip sequence there would be a predefined 30 second interval where an emotion was thought to be elicited in each clip.

**Figure 1:**
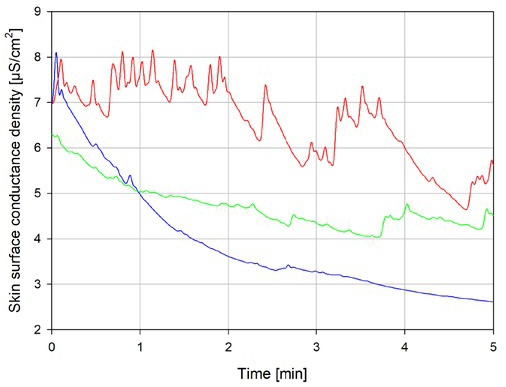
Example measurement of low frequency skin conductance. Person being highly stressed (red curve), moderately stressed (green curve), and totally relaxed (blue curve).

The electrodermal response monitoring was performed by using 22 Hz skin conductance measurements with the Sudologger 3 device (Biogauge A/S, Norway) [7]. The instrument applies a voltage of 50 mV rms and uses a three-electrode system with Ambu WhiteSensor 40554 electrodes (Ambu A/S, Denmark). An example of data measured with this technology can be seen in Figure 1, where the same person has been monitored during three epochs of different level of mental arousal/stress. From top to bottom, the person reported himself as being highly stressed (red curve), moderately stressed (green curve), and totally relaxed (blue curve). It is clear from the figure that in this case at least, the sympathetic sweat activity detected by the instrument correlates well with the self-experienced stress level.

**Figure 2:**
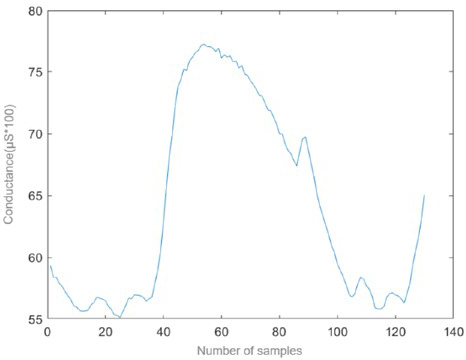
Conductance measurement. Example of data reported as disgust.

**Figure 3:**
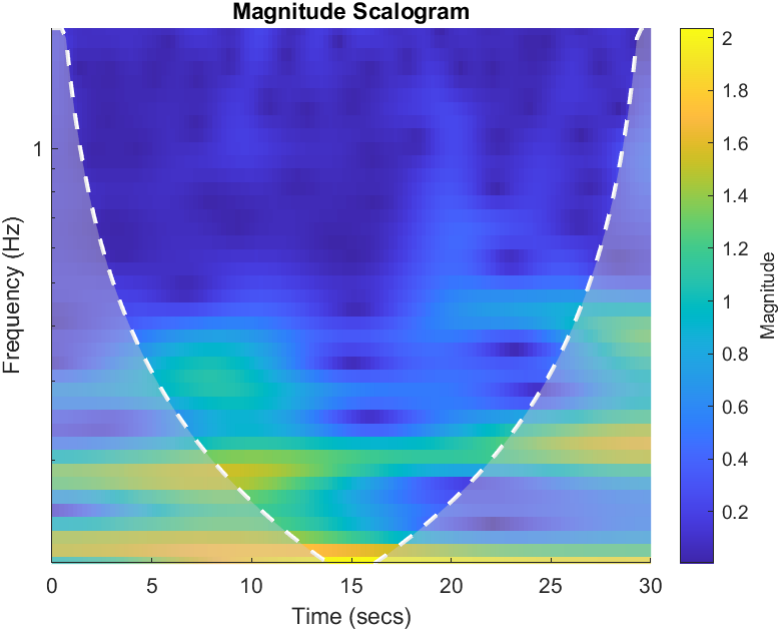
CWT treated conductance measurement. The same example (Figure 2) of data reported as disgust with CWT applied.

Data with obvious motion artifacts and noisy data due for instance to bad placement of the electrodes, were removed before the machine learning model was used. In order to make the data compatible with an image-based transfer-learning approach, the time-series were converted to images. The extracted thirty seconds of data for each clip for each test subject was then used to obtain a continuous wavelet transform (CWT) of the data with a Matlab program. The CWT was obtained using the analytic Morse wavelet with the symmetry parameter (gamma) equal to 3 and the time-bandwidth product equal to 60. The Matlab cwt-function uses 10 voices per octave. The minimum and maximum scales are determined automatically based on the energy spread of the wavelet in frequency and time. An example of the data extracted can be seen in Figure 2 and the corresponding CWT results can be seen in Figure 3.

All the processed data were then labelled and used in the machine learning model. We chose to test the VGG16, which is a commonly used convolutional neural network model [8].

### Informed consent

Informed consent has been obtained from all individuals included in this study.

### Ethical approval

The research related to human use has been complied with all relevant national regulations, institutional policies and in accordance with the tenets of the Helsinki Declaration, and has been approved by the authors’ institutional review board or equivalent committee.

## Results and data analysis

The experiment included ten test subjects and the data gathered can be seen in Table 1.

**Table 1:** Category of emotion and how many times the emotion was reported.

Emotion	Number of samples
Amusement	20
Anger	9
Disgust	24
Fear	5
Neutral	15
Sadness	26
Tenderness	1

All the data were cut down to thirty seconds of measurement resulting in a hundred-sample data set. An example of a sample gathered from the measurements and extracted into a thirty second cut, can be seen in Figure 2.

Following this extraction the CWT was applied to all the data and the imagery was labelled and set up as a data set for the image classification algorithm. An example, using the same data presented in Figure 2, of the CWT applied to the gathered data can be seen in Figure 3.

In Table 2, the results from the model using full EDA data sets with CWT applied, can be seen. This was the initial run of the VGG16 model with a random forest using the features. The Figure 5 shows, with the use of confusion matrices, that the data is poorly distributed among the different classes and the classifier wrongly classifies most data as sadness, giving sadness a low precision score and a noticeably higher recall score. Very little data is classified as anything other than sadness. Only amusement and disgust have any correct classification other than sadness.

**Figure 4:**
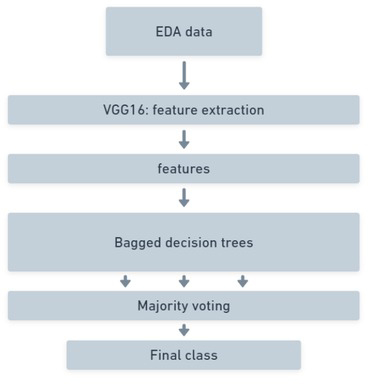
Architecture of the machine learning process.

**Figure 5:**
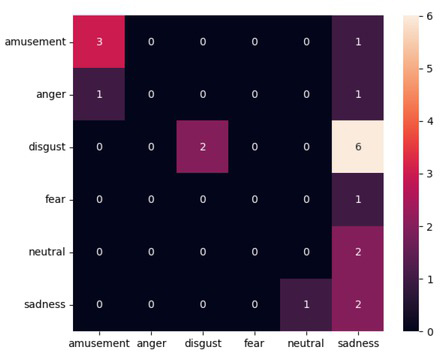
Confusion matrix for the first results produced by the model using the full dataset.

Having the CWT treated data the architecture showed in Figure 4, was practiced. For the whole of this project the test-train-split was 80/20. 80% of the data went to the training of the model, while 20% of the data was used as test data. The initial run of the model using 100 trees can be seen in Figure 5 and the more in depth results can be read from Table 2.

**Table 2:** Results using the CWT treated EDA data with test size = 20 and trees = 100.

test size = 20			trees = 100		

		precision	recall	f1-score	support
amusement		0.75	0.75	0.75	4
anger		0.00	0.00	0.00	2
disgust		1.00	0.25	0.40	8
fear		0.00	0.00	0.00	1
neutral		0.00	0.00	0.00	2
sadness		0.15	0.67	0.25	3

accuracy				0.35	20
macro avg		0.32	0.28	0.23	20
weighted avg		0.57	0.35	0.35	20

An alternation of the data set was tested out to see how the model performed with fewer categories to consider. Several alternations to the data set were done and categories removed. Seeing how most of the data was categorised as sadness it was interesting to see how the model did without this category and if the limiting of categories might help the performance of the model. The removal of categories was done for all the categories one by one and combinations like removing all the underrepresented categories leaving only four (or even three) categories and the corresponding data, made the data set even smaller, but also gave the classifying model fewer categories to consider.

**Figure 6:**
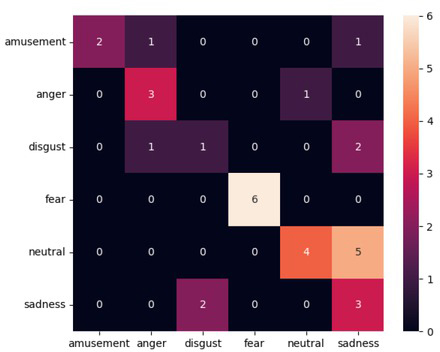
Confusion matrix for the first results produced by the model using the full data set with SMOTE data added.

After this was done, the addition of synthetic data was tried using the Synthetic Minority Over-sampling Technique (SMOTE). This was done with the Python SMOTE-API, which uses k-nearest neighbour to evaluate data within one category and makes copies that fall close to the original data. The addition of the synthetic data gives us more data to work with and the model gets to work with a more balanced data set. The same initial run, as presented above, can be seen in table 3 and Figure 6 with the addition of synthetic data. Now the model has 32 samples to work with instead of the original 20.

We see a more promising diagonal evolution of the classifying by the model and the results in Table 3 are far better than the results in Table 2. By adding the synthetic data, the model correctly classifies data for all the emotions instead of only the most supported ones.

**Table 3:** Results using the full CWT applied EDA dataset with synthetic data. The parameters are set to: test size = 20 and trees = 100.

test size = 20			trees = 100		

		precision	recall	f1-score	support
amusement		1.00	0.50	0.67	4
anger		0.60	0.75	0.67	4
disgust		0.33	0.25	0.29	4
fear		1.00	1.00	1.00	6
neutral		0.80	0.44	0.57	9
sadness		0.27	0.60	0.37	5

accuracy				0.59	32
macro avg		0.67	0.59	0.59	32
weighted avg		0.70	0.59	0.61	32

**Figure 7:**
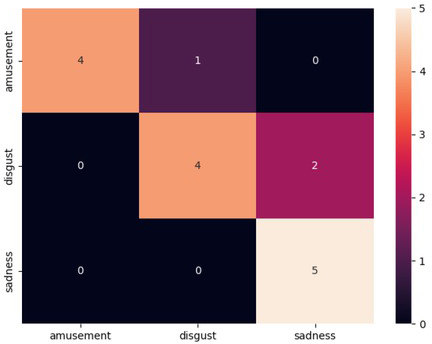
Confusion matrix for the results produced by the model using the three category data set with SMOTE data added.

Doing the same tinkering and alternation of the data set now only with the addition of synthetic data and a more balanced data set, there were clearly better results when the model did the image classification. Removing all the underrepresented categories and keeping the three most reported emotions provided a satisfactory result with the highest individual f1-scores for each category and a good accuracy score of 0.81. The results can be seen in Table 4 and Figure 7.

**Table 4:** Results using the 3 class CWT EDA data with synthetic data. Test size = 20 and trees = 300.

test size = 20			trees = 600		

		precision	recall	f1-score	support
amusement		1.00	0.80	0.89	5
disgust		0.80	0.67	0.73	6
sadness		0.71	1.00	0.83	5

accuracy				0.81	16
macro avg		0.84	0.82	0.82	16
weighted avg		0.84	0.81	0.81	16

## Discussion

It was harder to find a model based on EDA data to consider for transfer learning. Unfortunately, VGG16 is not trained on EDA data, but because it is trained on a large data set with many variations within the data, it was decided that it would be suitable to test the approach of transfer learning on EDA data.

Features extracted from the VGG16 model was used as input to a random forest classifier in order to handle small data sets. The tuneability of the model ended up being mainly its ability to change the number of trees used when classifying. The Python module sklearn.ensemble.RandomForestClassifier was used with (except for the number of trees) the default values for the parameters.

The initial over-classification of the sadness category points to one of two possibilities, either the sadness data is very varied within the class and the training of the model makes it easier to classify data as sadness, driven by the fact that the model has the most sadness data to work with. The other possibility is that the data, overall, is varied and the data presented as test data is hard for the model to classify. There seems to be a combination of these two.

The evidence of an unbalanced data set is obvious in the support column of the tables mentioned. Only one instance of fear is present in the test data and both neutral and anger have two instances. The effect of the unbalanced data is also reflected in the training data and can explain the poor results for these categories.

Within the existing data set there is a variance. Both in the data set and within the separate classes. This can be seen in table 2 where disgust has the most data to work with but the amusement category does better. Given that amusement has 20 instances and disgust have 24 they both have the same amount of data used for training the model. Such a difference in results can be explained by the variance within the class. Amusement seems to have the most unstable signals since the participants mostly moved from laughter, but the model recognizes this and the variance within the data set is then small. The same seems to relate to the disgust category as the other categories, wrongly categorized most of the time as sadness or the other better supported categories of emotions.

Even with this in mind, the model never performs below the random threshold for such a multiclass classification. With tenderness included in the data set, the threshold for the model to classify data at random is at 100*/*7 = 14.285 for accuracy and the poorest performance is at 0.35.

Removing outliers proved to be a challenge. Therefore the removal of categories was done to make the models work easier by providing fewer baskets to sort the data in. Starting by only removing tenderness proved fruitful in that the data was less wrongly categorized as sadness and, if not a lot, more spread out.

When we increase the number of trees, the model performed about the same as the best results with tenderness included, never going below the random threshold of 16.66, and now including a total of six categories.

The sadness data was, at one point, removed from the data set. This was done to counteract the models tendencies to wrongly categorize bigger parts of the test data as sadness and to see if the model would then perform better. This was not the case and the model performed worse, if we compare the different multiclass classifiers against each other. The effect of removing such a huge chunk of data only strengthens the argument and the reality that the data set is already on the smaller side. Without the sadness data the data set loses almost 1/4 of its total data. The model performs worse and the classes with the least amount of data gets wrongly classified as one of the other two classes with the most instances, amusement and disgust.

After running these and more tests on parameters and number of categories there was a clear need for more data. Due to limited data availability, the use of synthetic data was chosen as a solution. Figure 6 shows the results using synthetic data with the same data set and parameters as in Figure 5.

Introducing the synthetic data helped balance the data set right away and the results can be seen immediately by looking at Figure 6. Every category gets recognized and classified by the model and the data are more smoothly distributed. The introduction of the synthetic data has clearly made the model more confident in the groupings of the classes and is making the classification process easier. A sequence of tests shows that there are still fluctuations from the model when classifying for different parameters, which still means it struggles with the data set, its size and the data itself.

Having a more balanced data set made it possible to test out the same approaches as before. Removing the sadness data and limiting the data set to only four categories both showed better results than without the synthetic data, but slightly worse than the full data set if compared. For the standalone data set, where sadness and tenderness were removed, the accuracy score of 0.75 is promising. With the same number of trees there is also promise in that the model provides an f1-score of above 0.50 for all the categories. Looking closer there are still categories just barely getting over that 0.50 mark. On the other hand, we have the least supported categories, fear and anger, which perform very well.

Lastly, the categories that initially performed so poorly that they got a score of 0.00 across the board in the first, main run with the full data set (as can be seen e.g. in Table 2), were removed leaving a small data set and only three categories. The remaining three emotions had the most support of all, but the data set had now been shortened down by 30 data points. Figure 7 shows the increase in accuracy one would expect when increasing the number of trees for such a model. Reaching a peak at 600 trees with f1-scores of 0.89, 0.73 and 0.83 for amusement, disgust and sadness respectfully, making this the best individual f1-scores for all of the categories.

With these results, it seems that the model finds it hard to handle too many classes where the data is unbalanced and the variance is high both within the classes and the whole data set. This can be seen by the difference in scores achieved when the model is presented with a different modified data set where whole categories of data are taken out.

## Conclusion

The data and the model started working better together as the model was tweaked. There are of course many other methods that could have given some improvements of the results, and that should be investigated in future studies. One approach would be to reorganize the initial experiment and simplify it with fewer categories. Enlarging the data set would also be most beneficial, which is backed by the findings after applying synthetic data.

After expanding the data set with more data it is possible to test out other methods for classification, like support vector machines, logistic regression and K-nearest neighbors together with multiclass models such as one-vs-the-rest and one-vs-one. If there is enough data, a completely separate CNN could be set up. Exploring these alternatives, k-fold cross-validation can be used to compare the different methods and the different models can be combined to make predictions. This helps with variance and enhances generalization.

As mentioned in the theory, emotion elicitation using films is a very individual experience. Having a database sets a good standard for the experiment and the film clips have to have validity in terms of emotion elicitation. What happened through the experiment was that subjects who had seen the movies before, or even several times before, would often self-report a neutral state, leading to the neutral-state being one of the more reported states. But still, as the results show, this state is one of the hardest to interpret for the model and perhaps the least interesting for the bigger picture.
